# Polymicrobial biofilms in chronic rhinosinusitis: a scoping review

**DOI:** 10.1099/jmm.0.002104

**Published:** 2025-12-17

**Authors:** David Broderick, Kristi Biswas, Raymond Kim, Richard Douglas

**Affiliations:** 1 Department of Surgery, The University of Auckland, Auckland, New Zealand; 2 Te Whatu ora I Te Toka Tumai Auckland, Auckland City Hospital, Auckland, New Zealand

**Keywords:** antimicrobial resistance, bacterial interactions, biofilm, chronic rhinosinusitis (CRS), polymicrobial, review

## Abstract

**Introduction.** Biofilms have been implicated as a potential cause of chronic rhinosinusitis (CRS), with patients showing an increased prevalence of biofilms, likely contributing to antibiotic ineffectiveness in these individuals. In many environments, biofilms are polymicrobial, with interspecies interactions promoting bacterial survival and encouraging robust growth. Improvements in visualization techniques for biofilms have enabled species-specific identification, leading to a growing body of literature using these techniques and examining severity in different phenotypes of CRS.

**Gap Statement.** It is unclear whether sinus biofilms are typically poly- or monomicrobial, and if they are correlated with clinical severity in CRS.

**Aim.** We conducted a scoping review to determine how prevalent biofilms were in sinus tissue of patients with CRS. Furthermore, we correlated disease severity with the presence of biofilms.

**Methodology.** We searched PubMed, Scopus, Medline and Web of Science databases for all studies which directly visualized biofilms on tissue from patients with CRS. After screening 1,853 search results, 39 studies were included for analysis in this review.

**Results.** Patients with CRS had a higher prevalence of biofilms compared with controls. We found no significant difference in the proportion of biofilms detected across visualization techniques or based on CRS phenotyping. Fifteen studies reported disease severity by biofilm status; most reported greater severity in patients with biofilms, although only some were statistically significant. Nine studies used techniques capable of detecting polymicrobial biofilms, all of which found a subset of polymicrobial biofilms.

**Conclusion.** Our findings demonstrate an increased prevalence of biofilms in patients with CRS, which may correspond to increased disease severity. The evidence for biofilms being polymicrobial is compelling, although it is based on a small number of studies.

## Introduction

Biofilms are structures in which bacteria adhere to surfaces and secrete extracellular matrix proteins and saccharides, such as poly-*N*-acetylglucosamine [1] . The physiological differences between bacteria growing in biofilms, as opposed to planktonically, remain an active area of research. Notable features of biofilm-forming bacteria include reduced metabolic activity and increased horizontal gene transfer [2,3]. These characteristics can enhance bacterial survival after exposure to antibiotics, which may explain why biofilms are often associated with diseases that are resistant to antibiotic treatment, such as chronic rhinosinusitis (CRS) [2], chronic suppurative otitis media [4] and recurrent urinary tract infections [5].

Transcriptomic analysis of biofilms has shown that bacterial gene expression changes over time during biofilm development, with mature biofilms exhibiting distinct expression profiles compared with those still forming [6]. The specific bacterial strain significantly influences the genes expressed. However, certain genes, such as those coding for fibrinogen-binding proteins (fib), fibronectin-binding proteins (fnbA and fnbB) and intercellular adhesion (icaA), are commonly associated with biofilm formation across *Staphylococcus aureus* strains [7]. These genes are often upregulated in response to sub-inhibitory concentrations of antibiotics, which can promote biofilm formation [8,9]. Gene expression also varies between mono- and polymicrobial biofilms. For example, *Pseudomonas aeruginosa* has been shown to utilize metabolic by-products from *S. aureus* to enhance its own biofilm formation, increase antibiotic resistance and prevent the build-up of toxic compounds, such as acetoin [10]. In both environmental and clinical settings, biofilms are often polymicrobial – comprising two or more bacterial species in close association. This proximity facilitates synergistic interactions that can enhance biofilm stability and resistance [11] . As a result, polymicrobial biofilms may require either higher doses or more prolonged antibiotic treatment to be effectively treated [12]. Accordingly, determining whether single-species or polymicrobial biofilms dominate in specific diseases is critical to provide tailored therapies. Advances in biofilm visualization, including scanning electron microscopy (SEM) and confocal scanning laser microscopy (CSLM) with fluorescent *in situ* hybridization (FISH), have improved the detection of polymicrobial biofilms in clinical samples [13].

CRS is characterized by persistent inflammation of the paranasal sinus mucosa lasting more than 12 weeks [2]. First-line treatment typically involves saline irrigation, intranasal corticosteroids and oral antibiotics and steroids. However, these treatments often fail to provide sustained symptom relief, and so patients with CRS may require sinus surgery to remove inflamed tissue and improve sinus ventilation and drainage [2]. Studies of the role of biofilms in CRS have been ongoing since 2004 [14]. Previous systematic reviews from 2007 and 2011 found that while biofilms are associated with CRS, they do not occur in every case, and their role in disease pathology remains unclear [15,16]. In the years since, research has increasingly focused on the correlation between biofilms and CRS phenotypes, clinical severity and outcomes [17,18]. Defining clinical severity in CRS remains challenging. A recent systematic review highlighted that many studies use inconsistent criteria or even differing diagnostic definitions [19]. Furthermore, methods for biofilm identification have evolved over time. Techniques such as CSLM and FISH now allow for more precise identification of bacterial species and improved detection of polymicrobial biofilms [13]. Despite these advances, there is still no clear consensus on the optimal approach for identifying biofilms within the sinuses. Most studies rely on the visualization of bacterial clusters rather than confirming biofilm-specific behaviours [2]. Due to the heterogeneity within the literature, and the fragmented nature of research on polymicrobial biofilms in the sinuses, we deemed a scoping review to be the most appropriate approach, in accordance with recommended guidelines [20]. Accordingly, we conducted a scoping review to address three specific research questions: (i) How prevalent are biofilms in the sinuses of patients with CRS; (ii) Are biofilms in CRS associated with more severe disease? and (iii) Are biofilms in CRS polymicrobial?

## Methods

### Search protocol

This scoping review was conducted according to the Preferred Reporting Items for Systematic revies and Meta-Analyses(PRISMA) checklist [21]. We reviewed the International Prospective Register of Systematic Reviews to ensure no similar reviews were in progress. A literature search was performed on 9 July 2024 for publications since 2000 in Scopus, PubMed, Medline and Web of Science using the search terms (‘sinus’ OR ‘airway’ OR ‘respiratory’) AND (‘microbe’ or ‘bacteria’) AND ‘biofilm’. Search results were limited to English-language papers, original studies (reviews were excluded) and human-based studies. Only studies which directly visualized biofilms on patient-derived tissues were included.

### Selection of sources of evidence

Results were combined into a single spreadsheet with duplicates removed. Abstracts were screened for suitability, and all animal model and *in vitro* studies were removed. We also excluded studies in which the biofilm-forming capacity of clinical isolates was measured only *in vitro*, or where visualization was limited to abiotic surfaces with which the patient had contact, such as stents or surgical implements, rather than actual human tissue specimens.

### Bibliographic analysis

A bibliometric analysis was conducted using VOSviewer [22] on all search results, applying a keyword co-occurrence threshold of 40 instances. In parallel, Dimensions AI [23] was used to identify the most common research fields represented in the literature. Additionally, the number of publications per year was examined.

### Data charting

For the studies included in this review, population data, visualization methodology and outcome measures were extracted. Extracted population data included the number of patients with CRS, phenotypes, the proportion with previous sinus surgery and the presence and size of control and non-CRS disease cohorts. Extracted methodological data included the anatomical site of sample collection, visualization technique and grading of biofilms. Extracted outcome measures included the proportion and grade of biofilms in patient populations, and their correlation with clinical outcomes and severity measures. CRS phenotypes considered were polyp status, presence of eosinophilic mucus and allergy status. Visualization techniques were categorized by their ability to identify polymicrobial biofilms, and staining methods and species-specific probes were recorded where applicable. Only studies using multiple species-specific probes were considered capable of detecting polymicrobial biofilms. However, commentary on morphological differences in studies using non-specific probes was also recorded where present. Studies were scored by both the Strengthening the Reporting of Observational Studies in Epidemiology checklist and the National Institutes of Health Quality Assessment Tool [24,25].

### Biofilm image

**Fig. 1.**
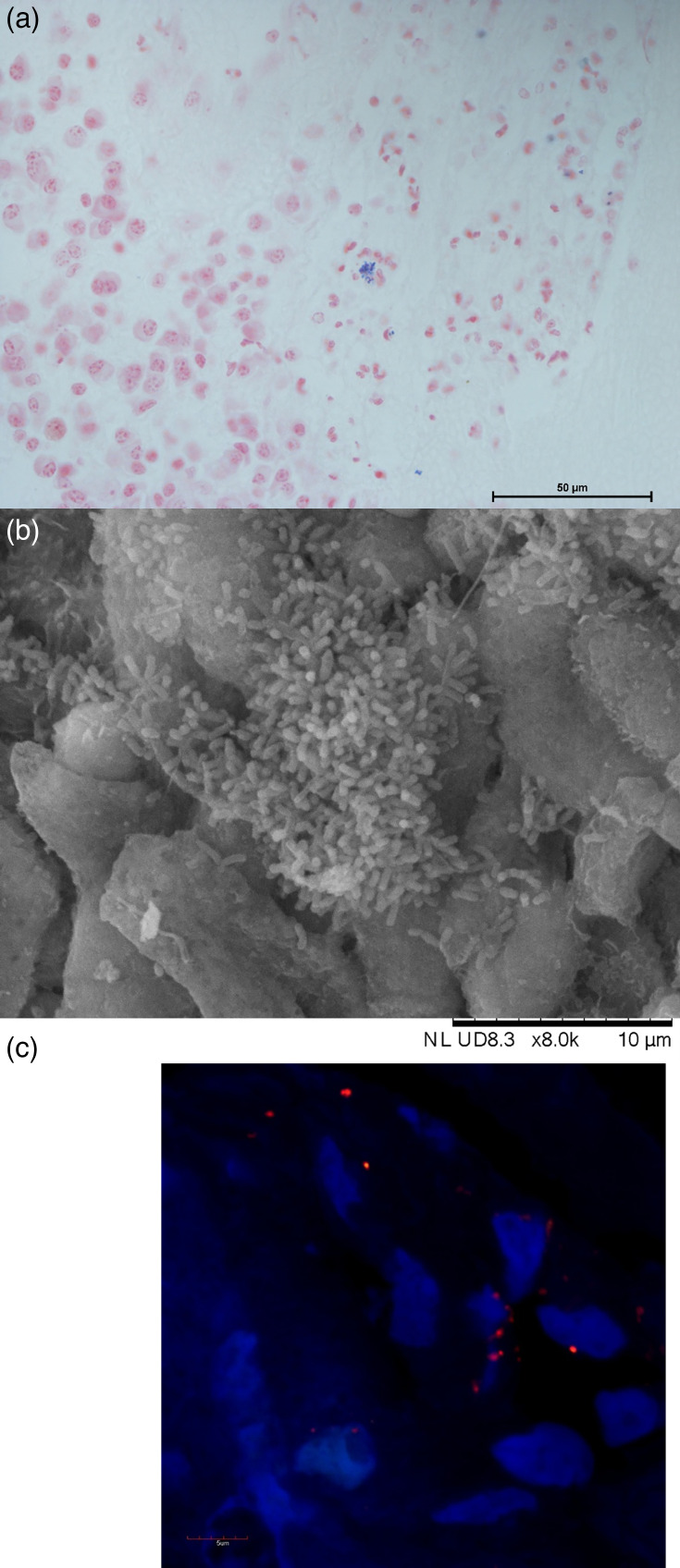
Representative images of biofilms visualized by different techniques. (a) Gram-positive-stained cells in the sinus tissue of patients with CRS at 63× magnification. The image was captured using a Leica DM1000 microscope. (b) SEM visualization of biofilms on human sinus tissue at 8,000× magnification. The image was captured using a TM3030Plus SEM (Hitachi Ltd., Tokyo, Japan). (c) FISH-stained *S. aureus* biofilm in sinus tissue of patients with cystic fibrosis and CRS. 4′,6-diamidino-2-phenylindole, DAPI (blue) stains all nucleic material, while *S. aureus* is identified using the Cyanine-3 labelled SAU (*Staphylococcus aureus*) probe (red) (Kempf *et al.*, 2000). The image was captured at 60× magnification using the Olympus FV1000 Confocal Laser Scanning Microscope (Olympus Corporation, Tokyo, Japan), with FluoView Software (version 4.2).

Previously unpublished images from studies within our research, and using protocols consistent with these, were taken to provide examples of different techniques used to visualize biofilms (Fig. 1). This included Gram staining in sinus tissue of patients with CRS at 63× magnification [26]; SEM on human sinus tissue at 8,000× magnification [2] and FISH-stained *S. aureus* biofilm in sinus tissue of patients with cystic fibrosis and CRS [27].

## Results

**Fig. 2.**
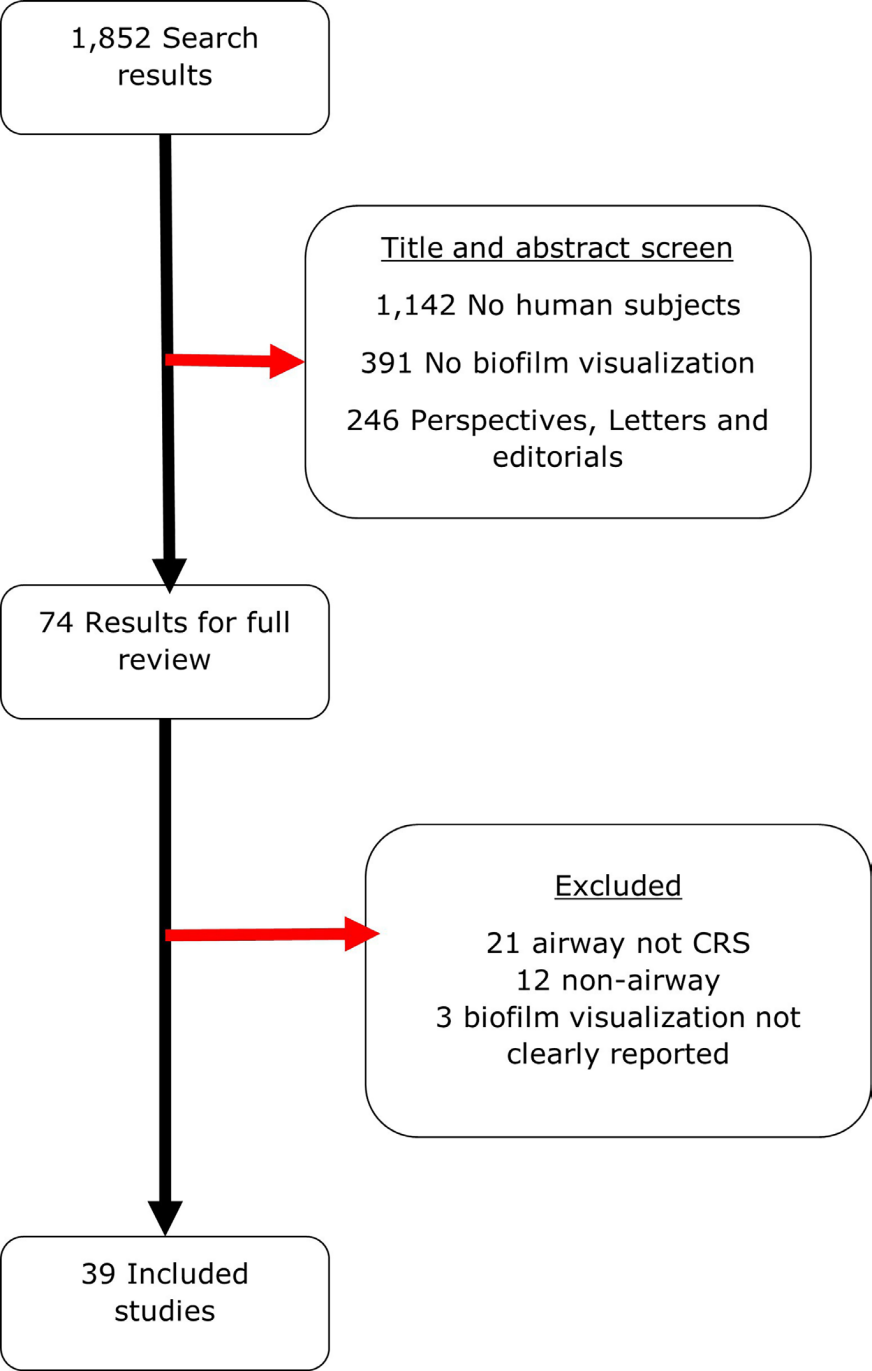
PRISMA diagram of study selection process.

The literature search identified 1,853 unique articles for title and abstract screening, 74 of which met our inclusion criteria for full review. Most studies were excluded due to a lack of human subjects or direct visualization of biofilms. After full review, 35 studies were excluded, primarily because they focused on airway conditions other than CRS. A complete list of exclusion criteria is provided in Fig. 2. Ultimately, 39 studies were included in data extraction, with full results for this reported in Table S2.

**Fig. 3.**
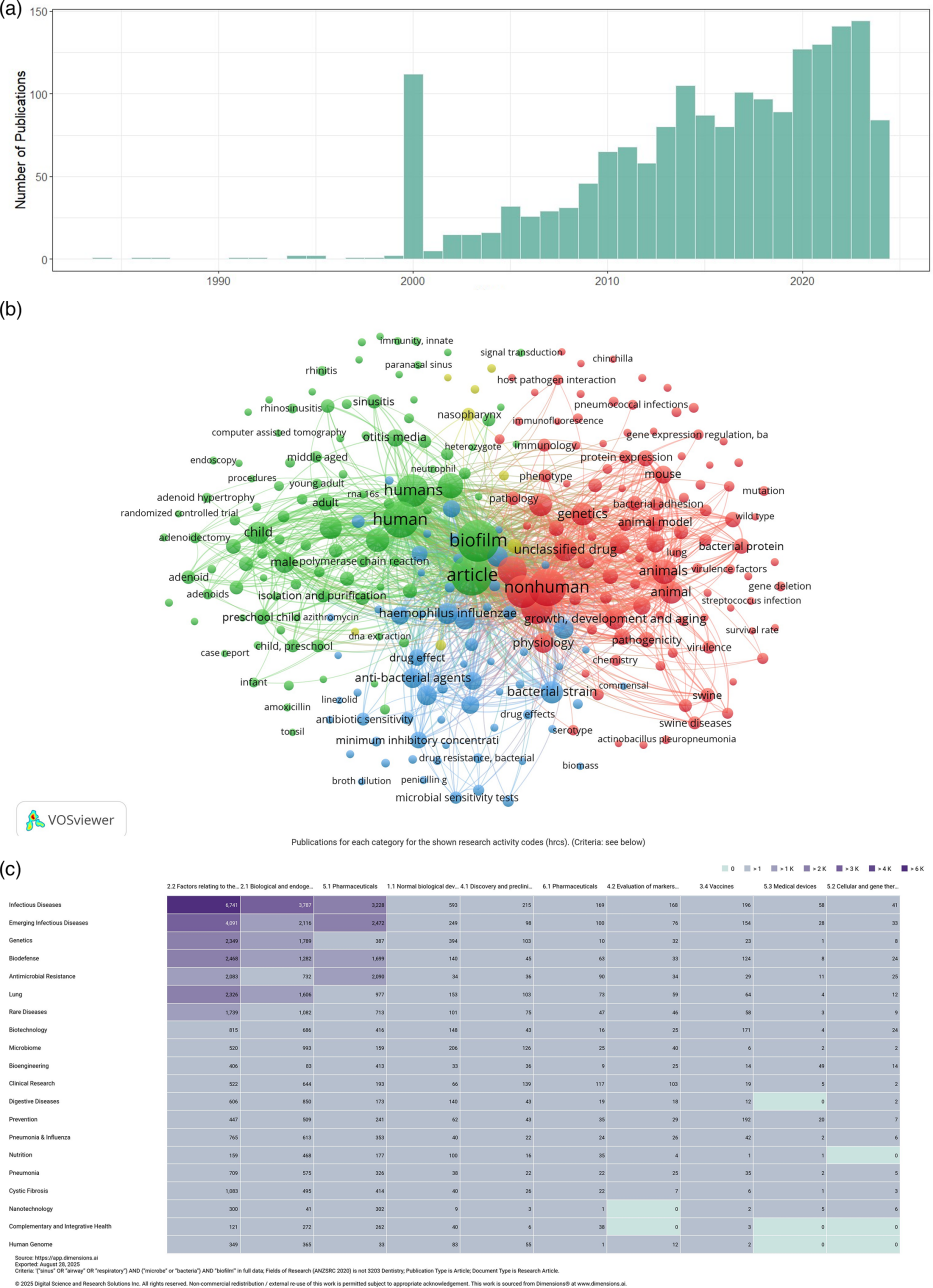
Bibliographic analysis of search results. (a) Number of search results by year, showing a growing number of publications each year, with the exception of 2000 – which is likely the result of increased indexing. (b) Keyword analysis of search results produced using VOSviewer, in which three clusters can be seen, reflecting the themes that VOSviewer's analysis has identified. (c) Grid analysis of the number of publications found by the initial search by year. The growing number of publications reflects the increasing interest in the topic of biofilms.

The trend in publications over time shows a gradual increase in the number of manuscripts published annually, indicating growing interest in this research area (Fig. 3a). Notable, a spike in publications around the year 2000 likely reflects technological advancements in publication databases [28,29]. The VOSviewer output identified three major keyword clusters, each representing distinct aspects of biofilm research. These included disease-related terms, such as rhinitis, adenoids and paranasal sinuses; biofilm physiology, including genetics, bacterial adhesion, virulence factors and pathogenicity; and drug development, represented by terms like drug effect, anti-bacterial agents and microbial sensitivity tests (Fig. 3b). These clusters were also reflected in the Dimension AI analysis, which categorized the literature primarily under ‘infectious disease’ and ‘emerging infectious disease’ (Fig. 3c). The most relevant research areas identified included factors related to the physical environment, biological and endogenous factors and pharmaceuticals.

### Methods to detect biofilms in CRS samples

A wide range of techniques were used to visualize biofilms across studies, including SEM, transmission electron microscopy (TEM), light microscopy with various staining methods (such as Gram staining, haemoxylin and eosin and Giesma staining) and CSLM, often using both universal and species-specific probes (Table 1, Fig. 1). While all techniques were employed across the studies, CSLM became more common in later studies, with BacLight being the most frequently used stain to differentiate live versus dead bacteria [30]. Additional CSLM staining methods targeted key sinus pathogens, including *S. aureus*, *P. aeruginosa*, *Haemophilus influenzae*, *Streptococcus pneumoniae* and universal fungal probes. Seven studies used these probes [30,36], and studies used only *S. aureus* probes [37,40]. Most studies did not apply grading or scoring criteria for biofilm detection. Of the eight that did, all used descriptions of bacterial density, but the grading methods were inconsistent. Finally, while many studies included a wash step to remove planktonic bacteria (49%), and nine SEM-based studies noted the presence of matrix components surrounding bacterial cells, only one study specifically targeted the visualization of extracellular matrix components [41].

**Table 1.** Included studies after study selection, with a summary of the visualization technique used and the species visualized, when available The total number of patients and controls, and the percentage in which biofilms were observed, were reported. For those papers which visualized multiple species, the evidence that might support the existence of polymicrobial biofilms is documented.

	Visualization technique	Patients with CRS, *n* (% biofilm positive)	Control patients, *n* (% biofilm positive)	Reporting of polymicrobial biofilm
Cryer *et al.*, 2004 [14]	SEM	16 (25%)		
Sanclement *et al.*, 2005 [59]	SEM, TEM	30 (80%)	4 (0%)	
Ramadan, 2005 [59]	SEM	5 (100%)		
Sanderson *et al.*, 2006 [31]	CSLM (FISH: SP, SA, HI, PA)	18 (78%)	5 (67%)	21.4% of identified biofilms were polymicrobial
Psaltis *et al.*, 2007 [60]	CSLM (BacLight)	38 (45%)	9 (0%)	
Healy *et al.*, 2008 [41]	CSLM (FISH: UF, SP, SA, HI, PA)	12 (75%)	3 (100%)	Most biofilms showed bacterial and fungal elements; close relationships between bacterial species were not shown
Galli *et al.*, 2008 [43]	SEM	24 (42%)	20 (0%)	Some biofilms were a mix of cocci and rods
Psaltis *et al.*, 2008 [42]	CSLM (BacLight)	40 (50%)	0 (%)	
Foreman *et al.*, 2009 [32]	CSLM (FISH: SA, PA, HI, UF)	50 (72%)	10 (%)	52.8% of biofilms had more than species detected
Dworniczek *et al.*, 2009 [61]	SEM, TEM	15 (47%)		
Tan *et al.*, 2010 [62]	CSLM (BacLight)	31 (39%)	11 (0%)	
Foreman *et al.*, 2010 [63]	CSLM (BacLight, FISH: SA, PA, HI)	20 (70%)		Some patients have multiple species detected as biofilms; it is unclear as to if these are mixed or multiple biofilms
Hochstim *et al.*, 2010 [64]	CSLM (BacLight), optical (H and E)	24 (62.5%)	10 (10%)	One patient had bacterial and fungal biofilm but in separate regions
Zernotti *et al.*, 2010 [65]	CSLM, optical (Gram-stain)	12 (17%)	10 (0%)	
Tóth *et al.*, 2011 [66]	Optical (H and E; Gram-stain)	50 (88%)	12 (0%)	25% of identified biofilms had fungal and bacteria identified
Wood *et al.*, 2011 [67]	CSLM (UB), optical (Giesma, Gram-stain)	18 (78%)	7 (43%)	
You *et al.*, 2011 [68]	SEM	93 (71%)	37 (0%)	
Jervis-Bardy *et al.*, 2011 [69]	CSLM (FISH: SA, PA, HI)	29 (55%)		Not reported
Calò *et al.*, 2011 [70]	SEM	24 (42%)		
Ragab *et al.*, 2012 [71]	SEM	22 (100%)	10 (0%)	All biofilms were identified as cocci
Li *et al.*, 2012 [72]	CSLM (BacLight)	27 (59%)	10 (0%)	
Tan, 2012 [37]	CSLM (FISH: SA)	17 (not reported)		
Karosi *et al.*, 2012 [73]	Optical (combination H and E and Gram-stain)	36 (81%)	8 (unclear)	13.4% of detected biofilms showed fungal and bacterial elements
Tan, 2012 [38]	CSLM (FISH: SA)	36 (81%)	5 (0%)	
Tan *et al.*, 2013 [39]	CSLM (FISH: SA)	51 (71%)		
Shields *et al.*, 2013 [74]	CSLM (FISH: universal bacterial), TEM	20 (not reported)		
Atay *et al.*, 2013 [17]	SEM	20 (80%)	15 (not reported)	
Jardeleza *et al.*, 2013 [40]	CSLM (FISH: SA)	17 (65%)	6 (0%)	
Arjomandi *et al.*, 2013 [33]	CSLM (FISH: HI, PA, UF, SA), optical (H and E)	20 (75%)	9 (0%)	Notes that polymicrobial biofilms were found
Foreman *et al.*, 2013 [32]	CSLM (FISH: HI, PA, UF, SA)	20 (80%)	5 (20%)	10% of biofilms were polymicrobial
Boase *et al.*, 2013 [44]	CSLM (FISH: SA, UF)	38 (50%)	6 (0%)	
Głowacki *et al.*, 2014 [18]	SEM	80 (41%)	33(%)	
Cantone *et al.*, 2014 [75]	Optical (Giesma)	84 (0%)		
Khosravi *et al.*, 2014 [35]	CSLM (FISH: HI, SP, SA, PA), SEM	15 (53%)	15 (0%)	82.4% of identified biofilms were polymicrobial (not specifically CRS)
Marcinkiewicz *et al.*, 2015 [76]	SEM	10 (30%)		
Mao *et al.*, 2015 [77]	SEM	24 (54%)		
Dlugaszewska *et al.*, 2016 [46]	SEM	30 (81%)	20 (88.2%)	
Di, 2018 [36]	CSLM (FISH: UF, SP, SA, HI, PA)	12 (64%)	5 (0%)	57.1% of identified biofilms were likely polymicrobial
Wang *et al.*, 2018 [78]	SEM	40 (54%)	23 (not reported)	
Jeican *et al.*, 2020 [79]	SEM	32 (43.7%)		

H and E, Hematoxylin and eosin staining; HI, *Haemophilus influenzae*; PA, *Pseudomonas aeruginosa*; SA, *Staphylococcus aureus*; SP, *Streptococcus pneumoniae*; UB, universal bacterial probe; UF, universal fungal probe.

### Prevalence of biofilms in CRS samples

**Fig. 4.**
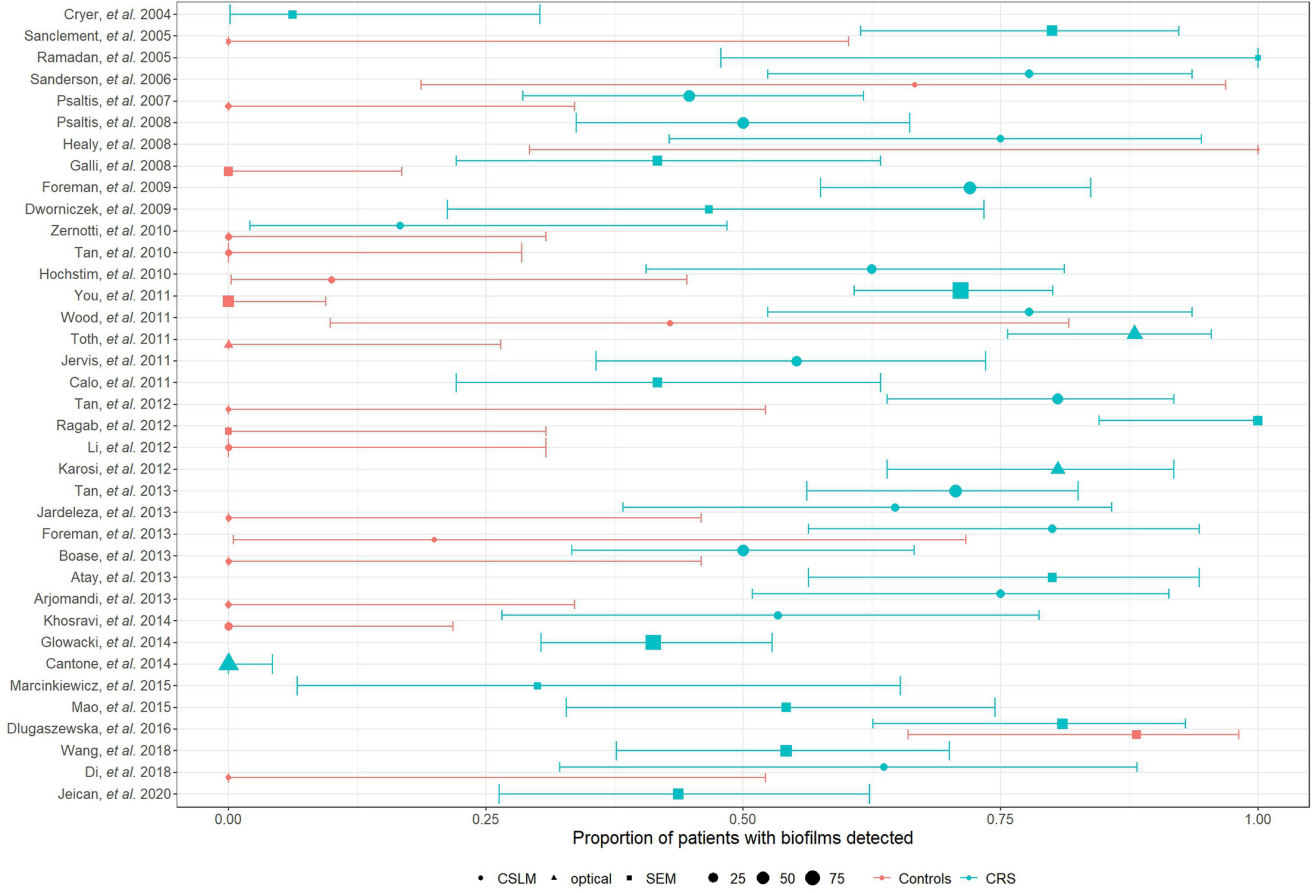
Prevalence of biofilms in control and CRS participants, grouped by study, with 95% confidence intervals determined by Poisson distribution. The shape of the data points represents the imaging methodology used in the study, while the size of the data point corresponds to the size of the study cohort. These data show a clear separation of controls and CRS participants with respect to biofilm prevalence across the majority of studies, with large confidence intervals likely a result of low sample size. Notable exceptions to this include Healy *et al.* [41], Dlugaszewska *et al.* [46] and Sanderson *et al.* [31], which have prevalence of biofilms in control patients similar to that of patients with CRS.

**Fig. 5.**
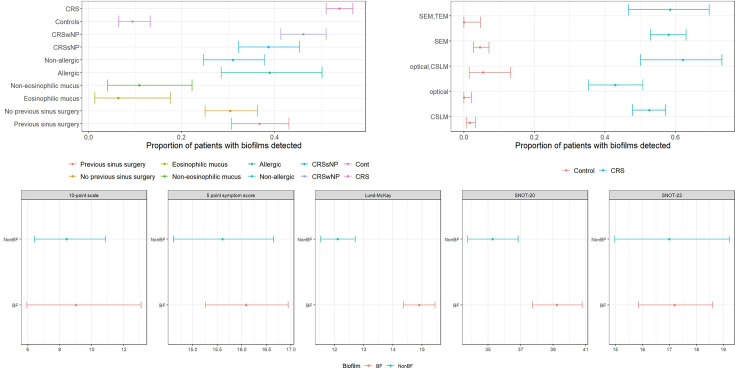
Synthesis of data from available summaries. (a) Proportion of patients who were biofilm-positive, with 95% confidence intervals determined by the Poisson distribution, grouped by clinical subgroup. (b) Proportion of controls and patients with CRS who were detected as biofilm-positive, with 95% confidence intervals determined by the Poisson distribution, grouped by imaging approach. There were no significant differences noted by imaging type, suggesting this may not play a large role in the prevalence of observed biofilms. (c) Summary of clinical severity measures by study, with scores shown with 95% confidence intervals determined by the Poisson distribution. The 10-point scale had a single contributing study; the Lund–McKay score had five contributing studies; SNOT-20 had three contributing studies; Sinonasal outcome test (SNOT-22) had a single contributing study; and symptom scores had three contributing studies. More information is available in the online supplementary table. Acronyms CRSwNP: CRS with nasal polyps; CRSsNP: CRS without nasal polyps; BF: biofilm positive cases

All but two studies identified biofilms in most patients with CRS, but prevalence was not uniform across patients or consistent between studies (Fig. 4), with some studies finding biofilm prevalence in controls similar to that of patients with CRS. Overall, proportionality tests revealed a significantly higher proportion of biofilms in patients with CRS compared with controls (*P*<0.001). Studies including both patients with CRS and controls consistently showed increased biofilm prevalence in patients with CRS. Among those with grading scores, CRS biofilms were more advanced or robust than those of controls when using the original paper’s assessment method (Fig. 5, Table S1, available in the online Supplementary Material).

### Association of biofilms with disease severity

Most studies (55%) included clinical variables or scoring systems, but these were heterogeneous across studies. Among these 17 studies, symptom scoring was the most common, with 14 studies using this approach, and four using the Sino-Nasal Outcome Test 20 (SNOT-20) questionnaire. Reporting of clinical severity by biofilm status was performed in 15 studies, with inconsistent results: four studies found no difference in clinical outcomes, ten reported worse outcomes in biofilm-positive patients, and one study provided unclear results. Lund–Mackay scores were higher in biofilm-positive patients in 70% of the studies where they were reported, though this was not always statistically significant. Symptom scoring showed worse results for biofilm-positive patients in 46% of reporting studies, although many of these results were not statistically significant (Table S1). Using a data synthesis approach, we consistently found that biofilm status suggested worse clinical outcomes for patients; however, only Lund–MacKay scores (nine studies) and SNOT-20 scores (four studies) showed significance (Fig. 5). Full severity data from the original papers are reported in Table S1.

The presence or absence of nasal polyps was the most common subtyping used in the included studies, with 20 studies reporting this detail. Eosinophilic mucus, allergy status and prior surgery were reported in 4, 13 and 15 studies, respectively. Differences by subgroup were not statistically significant, but CRS with polyps and patients with previous sinus surgery showed higher biofilm prevalence. Notably, one study reported a difference in the concordance of biofilm status with worse clinical outcomes by CRS phenotype, with biofilm status only corresponding to worse outcomes in patients without nasal polyps [42].

### Polymicrobial nature of biofilms in patients with CRS

Most biofilms characterized at the species level contained well-documented CRS pathogens, including *P. aeruginosa* and *S. aureus*. Other species detected were *H. influenzae* and *S. pneumoniae* (Table 1). Only nine studies used multiple-species probes, allowing for the identification of polymicrobial biofilms. The reporting of these findings was varied and brief; however, all identified some polymicrobial biofilms, with one study reporting that they made up 40% of detected biofilms. Several of these were bacterial biofilms with fungal elements. Additionally, a single study using electron microscopy noted the presence of biofilms containing bacterial cells with mixed morphologies, potentially indicating a polymicrobial community [43].

### Evaluation of literature quality

Using the NHI (National Heart Blood and Lung Institute) scoring system, 16 were scored fair and 24 as good, and the most common elements missing were sample-size justification, the lack of blinding or accounting for potential confounders. The average STROBE (Strengthening the Reporting of Observational Studies in Epidemiology) score was 16.68.

## Discussion

This review aligns with previous systematic reviews, highlighting that biofilms are more prevalent in patients with CRS than in controls [15,16]. Subsequent studies have allowed a more detailed investigation into the role of biofilms in clinical severity and pathology. While most studies report increased clinical severity in relation to biofilm status, there is variability in the measures used, and many studies have limited statistical power. Additionally, the role and diversity of bacterial communities in the sinuses have gained increasing recognition in recent literature [44]. This review acknowledges this trend by exploring the possibility of polymicrobial biofilms, which may account for a substantial number of biofilms found in patients with CRS. Importantly, this review is also limited by the scoping review process, in that most, if not all, of the included papers were unable to directly address our research question; instead, highlighting the need for more conclusive data in this space.

The visualization of biofilms remains a significant technical challenge, particularly due to the lack of clear diagnostic criteria and a grading system. Most studies did not directly image matrix components but included a washing step to remove planktonic bacteria. Some studies using SEM have reported the visualization of an extracellular matrix [41]. However, this complicates the interpretation of sinus images. SEM and TEM may struggle to distinguish bacteria from similar host-derived structures, while CSLM techniques currently lack biofilm-specific probes [2]. Notably, across different visualization methods, the proportion of biofilms in CRS remained consistent, suggesting that while technical limitations exist, they equally affect all imaging approaches.

While our review clearly shows biofilms are more prevalent in patients with CRS than in controls, it is important to consider that both a priori assumptions about relevant bacteria and preprocessing may influence this finding. Most studies had fewer controls than patients with CRS, and studies specifically focused on healthy controls have identified biofilms [45]. Mladina *et al.* [45] used SEM to visualize healthy biofilms, so it is possible that host-derived structures of a comparable size and shape were identified, or that the biofilm composition may differ from that typically observed in CSLM studies of patients with CRS. Additionally, studies included in this analysis, where biofilms were found and graded in healthy controls, noted that these biofilms were less robust [41,46] and may be more susceptible to wash steps.

The inconsistent prevalence and reporting of biofilms complicate associations with clinical severity or pathology. However, the consistent finding of worse clinical severity with the presence of biofilms (Fig. 5) suggests that biofilms warrant specific treatment strategies. Eradicating them may improve patient quality of life, even if it does not eliminate CRS. While biofilm formation is probably not the key pathogenic step in CRS, differences in biofilm prevalence across subtypes may indicate a role in disease progression and severity. Although not significantly different, we observed a higher prevalence of biofilms in CRS with polyps and in patients with recurrent surgery. These findings suggest that polyps may create an environment conducive to biofilm formation, or that biofilms may perpetuate disease. This may be due to surgical scar tissue [47] or disruption of the epithelial layer caused by an acute injury, such as a viral infection [48]. However, a single study reporting clinical severity by CRS type suggested that biofilms were only associated with increased severity in patients without polyps [42]. Future research should focus on the prevalence of biofilms across CRS phenotypes, and how this correlates with clinical severity measures, in order to address the current limitations in this analysis.

### Future perspectives

Our data suggest that polymicrobial biofilms may be present in a substantial proportion of patients with CRS. However, definitive conclusions are limited, as none of the included studies specifically focused on identifying polymicrobial biofilms. Nonetheless, growing evidence highlights the diversity of bacterial species present in CRS, alongside an expanding understanding of inter-species interactions observed in both *in vitro* and animal models [44,49]. Among airway-relevant bacteria, the most commonly studied interactions involve *S. aureus* and *P. aeruginosa*, with mixed-species models demonstrating enhanced biofilm robustness and increased antibiotic tolerance [50,51]. Similarly, mixed biofilms of *Candida albicans* and *S. aureus* have shown upregulation of resistance and virulence genes in both species [52]. Future studies should specifically address the polymicrobial nature, or lack thereof, in visualized biofilms. Techniques such as spatial genomics offer promising avenues for characterizing biofilm composition without requiring a priori assumptions about the bacterial species present. These approaches also enable the detection of gene transcription related to biofilm formation, antibiotic resistance and other virulence factors [53], helping to determine whether the synergistic interactions observed in wound models are also relevant in CRS.

The presence of biofilms in upper-airway disease has spurred the development of anti-biofilm therapeutics. These therapies are typically tested *in vitro* on monoculture biofilms; however, recent studies using polymicrobial biofilms in lower airway models found that polymicrobial biofilms exhibit greater resistance and, at certain doses, only eradicate specific species and allow others to overgrow [12]. Consideration of both overall biofilm prevalence and the presence of polymicrobial biofilms will be important when conducting power calculations and determining the required sample size to demonstrate therapeutic effectiveness. Our findings provide sufficient evidence to warrant further investigation into the concentrations of novel therapeutics needed to fully eradicate polymicrobial biofilms, as well as their potential impact on bacterial community composition. For example, *S. pneumoniae*, commonly identified in CRS microbiome studies [49], has been shown to form polymicrobial biofilms [54] and can reduce reactive oxygen species (ROS). Several antimicrobial products use the ROS strategy to target biofilms [55,56]. It is therefore plausible that the use of such biocides could increase the abundance of *S. pneumoniae*, which is associated with severe lower respiratory tract infections. This possibility warrants the need to investigate the impacts of novel therapeutic approaches on the microbiome [57,58].

## Conclusion

We have completed an updated review on the role of biofilms in CRS, which acknowledges the increased awareness of microbial diversity in the airways by considering the possibility of polymicrobial biofilms. Additionally, by analysing more recent publications, we have considered in greater detail the role of biofilms in CRS subtypes and disease severity. We found that most patients with CRS show the presence of biofilms, and their presence typically correlates with worse clinical symptoms, with only limited differences between CRS subtypes. Finally, while evidence remains limited, we identified that polymicrobial biofilms exist in a subset of patients with CRS with biofilms, which has important implications for the testing and clinical use of novel biofilm-eradication therapies.

## Supplementary material

10.1099/jmm.0.002104 Uncited Fig. S1.

10.1099/jmm.0.002104 Uncited Fig. S2.

## Abbreviations

CRS: chronic rhinosinusitis
CRSsNP: CRS sans nasal polyps
CRSwNP: CRS with nasal polyps
CSLM: confocal scanning laser microscopy
FISH: fluorescent *in situ* hybridization
ROS: reactive oxygen species
SEM: scanning electron microscopy
SNOT-20: Sino-Nasal Outcome Test 20
TEM: transmission electron microscopy
